# What are the differences in the outcome of laparoscopic axial (I) versus paraesophageal (II–IV) hiatal hernia repair?

**DOI:** 10.1007/s00464-017-5612-z

**Published:** 2017-06-08

**Authors:** F. Köckerling, Y. Trommer, K. Zarras, D. Adolf, B. Kraft, D. Weyhe, R. Fortelny, C. Schug-Paß

**Affiliations:** 1 Department of Surgery and Center for Minimally Invasive Surgery, Academic Teaching Hospital of Charité Medical School, Vivantes Hospital, Neue Bergstrasse 6, 13585 Berlin, Germany; 2 Department of General, Visceral and Minimally Invasive Surgery, Helios Hospital, Campus 6, 38518 Gifhorn, Germany; 3 Department of Visceral, Minimally Invasive and Oncologic Surgery, Marien Hospital, Rochusstrasse 2, 40479 Düsseldorf, Germany; 4 StatConsult GmbH, Halberstädter Strasse 40 A, 39112 Magdeburg, Germany; 5 Department of General and Visceral Surgery, Diakonie Hospital, Rosenbergstrasse 38, 70176 Stuttgart, Germany; 6 Department of General and Visceral Surgery, Pius Hospital, University Hospital of Visceral Surgery, Georgstrasse 12, 26121 Oldenburg, Germany; 7 Department of General, Visceral and Oncologic Surgery, Wilhelminenhospital, Montleartstrasse 37, 1160 Vienna, Austria

**Keywords:** Hiatal hernia, Fundoplication, Hiatoplasty, Axial hiatal hernia, Paraesophageal hiatal hernia

## Abstract

**Introduction:**

Comparison of elective laparoscopic repair of axial vs paraesophageal hiatal hernias reveals relevant differences in both the patient collectives and the complexity of the procedures.

**Materials and methods:**

The present uni- and multivariable analysis of data from the Herniamed Registry compares the outcome for 2047 (67.3%) (type I) axial with 996 (32.7%) (types II–IV) paraesophageal primary hiatal hernias following laparoscopic repair.

**Results:**

Compared with the patients with axial hiatal hernias, patients with paraesophageal hiatal hernia were nine years older, had a higher ASA score (ASA III/IV: 34.8 vs 13.7%; *p* < 0.001), and more often at least one risk factor (38.8 vs 21.4%; *p* < 0.001). This led in the univariable analysis to significantly more general postoperative complications (6.0 vs 3.0%; *p* < 0.001). Reflecting the greater complexity of the procedures used for laparoscopic repair of paraesophageal hiatal hernias, significantly higher intraoperative organ injury rates (3.7 vs 2.3%; *p* = 0.033) and higher postoperative complication-related reoperation rates (2.1 vs 1.1%; *p* = 0.032) were identified. Univariable analysis did not reveal any significant differences in the recurrence and pain rates on one-year follow-up. Multivariable analysis did not find any evidence that the use of a mesh had a significant influence on the recurrence rate.

**Conclusion:**

Surgical repair of paraesophageal hiatal hernia calls for an experienced surgeon as well as for corresponding intensive medicine competence because of the higher risks of general and surgical postoperative complications.

Four anatomic patterns of hiatal hernia can be recognized. Axial or sliding (type I) hernia, in which the gastroesophageal junction migrates into the thorax, is the most common type of hiatal hernia (95%) and may predispose to gastroesophageal reflux [1]. Type II represents a true paraesophageal hernia with herniation of the gastric fundus anterior to a normally positioned esophagogastric junction [1]. Type III, with both elements of types I and II hiatal hernia, tends to be large with more than 50% of the stomach within the mediastinal sac [1]. In type IV hernias, the stomach, sometimes with other viscera such as the colon or spleen, migrates completely in the hernia sac, which may result in an “upside-down stomach” [1]. Patients with an axial/sliding or type I hernia and long-term treatment of gastroesophageal reflux disease and continuous reduced quality of life, persistent troublesome symptoms, and/or progression of disease despite adequate proton pump inhibitor therapy in dosage and intake are the best candidates for surgery [2]. Although paraesophageal hernias types II–IV account for only 5% of all hiatal hernias, their detection is important because of potentially life-threatening complications, such as obstruction, acute dilatation, perforation, or bleeding of the stomach mucosa [1]. In essence, no conventional options are available for the treatment of paraesophageal hernia, so surgical repair is recommended for relief of symptoms [1].

Laparoscopic hiatal hernia repair is as effective as open transabdominal repair, with a reduced rate of perioperative morbidity and with shorter hospital stays. It is the preferred approach for the majority of hiatal hernias [3–6]. Laparoscopic posterior fundoplication is given preference over laparoscopic anterior fundoplication due to a lower recurrence rate [7] in the treatment of gastroesophageal reflux disease. Thirteen randomized controlled trials with 1564 patients showed for Toupet versus Nissen fundoplication significantly lower rates of adverse results involving dysphagia, gas-bloat syndrome, inability to belch, and reoperation due to severe dysphagia [8, 9]. Mesh application should be considered for large hiatal hernia repair because it reduces recurrences, at least in the midterm. Overall, procedure-related complications and mortality do not seem to be increased despite potential mesh-associated complications [10–17].

In the literature, there is only one publication with a large case series which compares the patient collective, treatment, and the outcome of laparoscopic repair of type I hiatal hernias with those of paraesophageal hiatal hernias (types II–IV) [18]. In that study, most of the complications occurred in patients with paraesophageal compared with axial hernia (10 vs 1%, respectively) [18]. This variation reflects significant differences between patients with axial hiatal hernia, and gastroesophageal reflux disease, and those with paraesophageal hernia; it also highlights the increased complexity of the laparoscopic repair procedure used for paraesophageal hernia [18]. Based on data from the Herniamed Hernia Registry, this paper now explores the differences between these patients in terms of demographic characteristics, treatment, and outcome.

## Materials and methods

**Fig. 1:**
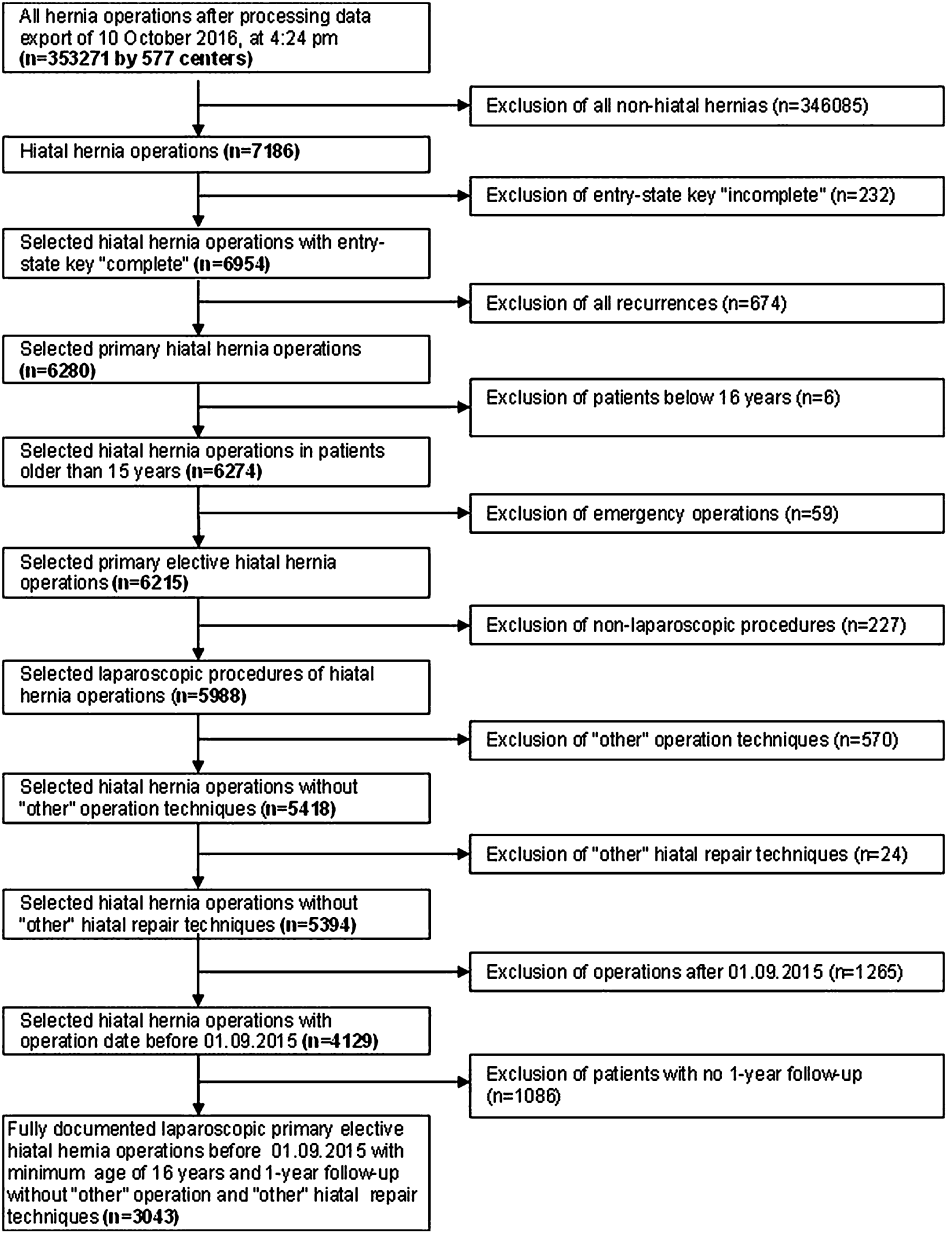
Flowchart of patient inclusion

The Herniamed quality assurance study is a multicenter, internet-based hernia register [19] into which 577 participating hospitals and surgeons engaged in private practice (Herniamed Study Group) in Germany, Austria, and Switzerland (status: October 10, 2016) have entered data prospectively on their patients who had undergone routine surgery and signed an informed consent agreeing to participate. As part of the information provided to patients regarding participation in the Herniamed Quality Assurance Study, all patients are informed that the treating hospital would like to be informed about any problems occurring after the operation and that the patient has the opportunity to attend for clinical examination. All postoperative complications occurring up to 30 days after surgery are recorded. On one-year follow-up, postoperative complications are once again reviewed when the general practitioners and patients complete a questionnaire. On one-year follow-up, general practitioners and patients are also asked about any recurrent symptoms, pain at rest, pain on exertion, and chronic pain requiring treatment. If recurrent symptoms or chronic pain are reported by the general practitioners or patients, patients can be requested to attend for clinical examination or radiologic tests. A recent publication has provided impressive evidence of the role of patient-reported outcomes in hernia surgery [20]. The present analysis compares the prospective data collected for all patients with a hiatal hernia (types I–IV) and laparoscopic repair. Inclusion criteria were minimum age of 16 years, primary elective laparoscopic operation, fundoplication or fundophrenicopexy, and availability of data on one-year follow-up. In total, 3043 patients were enrolled from 197 participating institutions with mean number of 15.4 (range 1–199) cases between September 1, 2009 and September 1, 2015 (Fig. 1). Of these patients, 2047 (67.3%) had an axial/sliding (type I) and 996 (32.7%) a paraesophageal (types II–IV) hiatal hernia (Table 1). No details of the diagnostic method used for classification of hernia type were included in the registry. The demographic parameters included age (years), gender, symptoms, ASA score (I, II, III, IV), body mass index (BMI) (kg/m^2^), and risk factors (COPD, diabetes, aneurysms, cortisone, immunosuppression, etc.). Risk factors were dichotomized, i.e., “yes” if a risk factor was positive and “no” otherwise.

**Table 1:** Distribution of cases based on hiatal hernia type

Type	*N*	%
Axial I	2047	67.3
Paraesophageal II	263	8.6
Mixed III	279	9.2
Upside-down IV	454	14.9
Total	3043	100

The second group of categorical influence variables reflecting surgery-related parameters included defect size, operation technique (Toupet vs Nissen vs fundophrenicopexy), and hiatoplasty (suture vs mesh vs suture and mesh).

The dependent variables were intra- and postoperative complication rates, complication-related reoperation rates, recurrence rates and rates of pain at rest, pain on exertion, and chronic pain requiring treatment.

All analyses were performed with the software SAS 9.4 (SAS Institute Inc. Cary, NC, USA) and intentionally calculated to a full significance level of 5%, i.e., they were not corrected in respect of multiple tests, and each *p* value ≤0.05 represents a significant result. To discern differences between the groups in unadjusted analyses, Fisher’s exact test was used for categorical outcome variables, and the robust *t* test (Satterthwaite) for continuous variables. To rule out any confounding of data caused by different patient characteristics, the results of univariable analyses were verified via multivariable analyses in which, in addition to hiatal hernia type, other influence parameters were simultaneously reviewed.

To access influence factors in multivariable analyses, the binary logistic regression model for dichotomous outcome variables was used. Estimates for odds ratio (OR) and the corresponding 95% confidence interval based on the Wald test were given. For influence variables with more than two categories, all pairwise odds ratios were given. For age (years), the 10-year OR estimate, for BMI (kg/m^2^), the five-point OR, and, for defect size, the ten-point OR estimate were given. For the procedure time (min) and hernia defect size (cm^2^), a logarithmic transformation was applied and re-transformed mean values and ranges specified. The results of multivariable analyses are presented in tabular form, sorted by descending impact.

## Results

### Univariable analyses

Patients with axial hiatal hernia (type I) and reflux disease compared with patients with paraesophageal hiatal hernia (types II–IV) were on average more than nine years younger, had a somewhat lower BMI, markedly shorter procedure time, and smaller hernia defects (Table 2).

**Table 2:** Comparison of mean age, mean BMI, mean procedure time, and mean defect size between axial and paraesophageal hiatal hernia types

		Type I	Types II–IV	*p*
Age (years)	Mean ± STD	55.4 ± 14.0	65.0 ± 12.5	<.001
BMI	Mean ± STD	27.7 ± 4.3	28.7 ± 4.8	<.001
Duration of procedure (min)^a^	MW (range)	83.0 (81.5; 84.6)	104.4 (102.8; 106.0)	<.001
Defect size (cm^2^)^a^	MW (range)	12.6 (10.5; 14.8)	21.5 (19.2; 23.7)	<.001

^a^Logarithmic transformation; indication of re-transformed mean and range of dispersion (mean-STD; mean + STD)

As regards the axial hiatal hernias (type I), Toupet fundoplication (56.2 vs 41.0%; *p* < 0.001) as well as hiatoplasty with suture alone were performed more often (81.5 vs 64.1%; *p* < 0.001) (Table 3). Besides, axial hiatal hernia was associated with lower ASA scores and a greater number of male patients (Table 3). On the other hand, for the paraesophageal hiatal hernias (types II–IV), more cases of fundophrenicopexy (19.5 vs 2.5%; *p* < 0.001) and of hiatal closure with suture and mesh (35.2 vs 17.7%; *p* < 0.001) were observed (Table 3). For the paraesophageal hernias (types II–IV), higher ASA scores (ASA III/IV: 34.8 vs 13.7%; *p* < 0.001) as well as more female patients (67.2 vs 56.2%; *p* < 0.001) were identified. Besides, the proportion of patients with at least one risk factor was significantly higher for paraesophageal hernias at 30.8 vs 21.4% (*p* < 0.001). In terms of symptoms, only reflux (89.3 vs 66.0%; *p* < 0.001) was more common for axial hiatal hernias (Table 3).

**Table 3:** Comparison of demographic parameters, risk factors, and surgery-related parameters between axial and paraesophageal hiatal hernia types

	Type I		Types II–IV		*p*
	*n*	%	*n*	%	
Procedure					
Fundophrenicopexy	51	2.49	194	19.48	<.001
Nissen	845	41.28	394	39.56	
Toupet	1151	56.23	408	40.96	
Hiatal repair technique					
Suture	1669	81.53	638	64.06	<.001
Suture and mesh	363	17.73	351	35.24	
Mesh	15	0.73	7	0.70	
ASA score					
I	464	22.67	88	8.84	<.001
II	1302	63.61	561	56.33	
III	277	13.5	339	34.0	
IV	4	0.20	8	0.80	
Gender					
Male	898	43.87	327	32.83	<.001
Female	1149	56.13	669	67.17	
Risk factor					
Total					
Yes	437	21.35	307	30.82	<.001
No	1610	78.65	689	69.18	
COPD					
Yes	168	8.21	137	13.76	<.001
No	1879	91.79	859	86.24	
Diabetes					
Yes	76	3.71	72	7.23	<.001
No	1971	96.29	924	92.77	
Aortic aneurysm					
Yes	5	0.24	8	0.80	0.036
No	2042	99.76	988	99.20	
Immunosuppression					
Yes	7	0.34	10	1.00	0.034
No	2040	99.66	986	99.00	
Corticoids					
Yes	20	0.98	19	1.91	0.039
No	2027	99.02	977	98.09	
Smoking					
Yes	162	7.91	58	5.82	0.037
No	1885	92.09	938	94.18	
Coagulopathy					
Yes	13	0.64	16	1.61	0.015
No	2034	99.36	980	98.39	
Antiplatelet medication					
Yes	62	3.03	68	6.83	<.001
No	1985	96.97	928	93.17	
Anticoagulation therapy					
Yes	21	1.03	15	1.51	0.284
No	2026	98.97	981	98.49	
Symptoms					
Reflux					
Yes	1827	89.25	657	65.96	<.001
No	220	10.75	339	34.04	
Regurgitation					
Yes	491	23.99	275	27.61	0.033
No	1556	76.01	721	72.39	
Dysphagia					
Yes	392	19.15	454	45.58	<.001
No	1655	80.85	542	54.42	
Pain					
Yes	763	37.27	484	48.59	<.001
No	1284	62.73	512	51.41	
Anemia/bleeding					
Yes	81	3.96	212	21.29	<.001
No	1966	96.04	784	78.71	
Affection of lung					
Yes	163	7.96	159	15.96	<.001
No	1884	92.04	837	84.04	

On overall assessment of the intraoperative complication rates no difference was detected between the axial (type I) and paraesophageal hiatal hernias (types II–IV) (Table 4). However, organ injuries were seen significantly more often with paraesophageal hiatal hernias (types II–IV) (3.7 vs 2.3%; *p* = 0.033).

**Table 4:** Comparison of intraoperative, postoperative, and general complications and 1-year follow-up outcome between axial and paraesophageal hiatal hernia types

	Type I		Types II–IV		*p*
	*n*	%	n	%	
Intraoperative complications					
Total					
Yes	60	2.93	41	4.12	0.105
No	1987	97.07	955	95.88	
Intraop.: bleeding					
Yes	28	1.37	14	1.41	1.000
No	2019	98.63	982	98.59	
Injuries					
Total					
Yes	47	2.30	37	3.71	0.033
No	2000	97.70	959	96.29	
Esophagus					
Yes	1	0.05	0	0.00	1.000
No	2046	99.95	996	100.0	
Stomach					
Yes	2	0.10	5	0.50	0.042
No	2045	99.90	991	99.50	
Bowel					
Yes	0	0.00	1	0.10	0.327
No	2047	100.0	995	99.90	
Liver					
Yes	8	0.39	3	0.30	1.000
No	2039	99.61	993	99.70	
Spleen					
Yes	8	0.39	7	0.70	0.274
No	2039	99.61	989	99.30	
Vessel					
Yes	3	0.15	2	0.20	0.665
No	2044	99.85	994	99.80	
Others (pleura opening, diaphragm injury)					
Yes	26	1.27	21	2.11	0.086
No	2021	98.73	975	97.89	
Postoperative complications (Clavien–Dindo classification grades I–III)					
Total					
Yes	24	1.17	20	2.01	0.076
No	2023	98.83	976	97.99	
Bleeding					
Yes	3	0.15	5	0.50	0.123
No	2044	99.85	991	99.50	
Esophageal perforation					
Yes	10	0.49	5	0.50	1.000
No	2037	99.51	991	99.50	
Infection					
Yes	3	0.15	6	0.60	0.067
No	2044	99.85	990	99.40	
Stomach perforation					
Yes	5	0.24	0	0.00	0.180
No	2042	99.76	996	100.0	
Wound healing disorder					
Yes	3	0.15	6	0.60	0.067
No	2044	99.85	990	99.40	
Ileus					
Yes	0	0.00	2	0.20	0.107
No	2047	100.0	994	99.80	
General complications					
Total					
Yes	61	2.98	60	6.02	<.001
No	1986	97.02	936	93.98	
Fever					
Yes	6	0.29	7	0.70	0.137
No	2041	99.71	989	99.30	
Urinary voiding problems					
Yes	4	0.20	5	0.50	0.163
No	2043	99.80	991	99.50	
Diarrhea					
Yes	1	0.05	1	0.10	0.548
No	2046	99.95	995	99.90	
Gastritis					
Yes	1	0.05	1	0.10	0.548
No	2046	99.95	995	99.90	
Thrombosis					
Yes	2	0.10	0	0.00	1.000
No	2045	99.90	996	100.0	
Pulmonary embolism					
Yes	1	0.05	3	0.30	0.106
No	2046	99.95	993	99.70	
Pleural effusion					
Yes	10	0.49	17	1.71	0.001
No	2037	99.51	979	98.29	
Pneumonia					
Yes	6	0.29	12	1.20	0.004
No	2041	99.71	984	98.80	
COPD (clinical exacerbation)					
Yes	7	0.34	7	0.70	0.251
No	2040	99.66	989	99.30	
Cardiac insufficiency					
Yes	4	0.20	10	1.00	0.003
No	2043	99.80	986	99.00	
Coronary heart disease					
Yes	5	0.24	4	0.40	0.486
No	2042	99.76	992	99.60	
Myocardial infarction					
Yes	1	0.05	2	0.20	0.251
No	2046	99.95	994	99.80	
Renal insufficiency					
Yes	2	0.10	0	0.00	1.000
No	2045	99.90	996	100.0	
Hypertensive crisis					
Yes	3	0.15	4	0.40	0.226
No	2044	99.85	992	99.60	
Complication-related reoperation (Clavien–Dindo classification grade III)					
Yes	22	1.07	21	2.11	0.032
No	2025	98.93	975	97.89	
Recurrence on 1-year follow-up					
Yes	105	5.13	40	4.02	0.204
No	1942	94.87	956	95.98	
Pain on exertion on 1-year follow-up					
Yes	222	10.85	102	10.24	0.661
No	1825	89.15	894	89.76	
Pain at rest on 1-year follow-up					
Yes	180	8.79	86	8.63	0.945
No	1867	91.21	910	91.37	
Pain requiring treatment on 1-year follow-up					
Yes	166	8.11	71	7.13	0.387
No	1881	91.89	925	92.87	

As regards the postoperative surgical complications, no significant difference was detected between the axial (type I) and paraesophageal hiatal hernias (types II–IV). However, more complication-related reoperations (Clavien–Dindo classification grade III) were noted for paraesophageal compared with axial hernias (2.1 vs 1.1%; *p* = 0.032) (Table 4). The main reasons for this were esophageal and gastric injuries, secondary bleeding, and abscesses.

For the general postoperative complications, a highly significant difference to the disadvantage of the paraesophageal hernias (types II–IV) was detected at 6.0 vs 3.0% (*p* < 0.001) (Table 4). Since one-year follow-up was a precondition for patient selection, analysis did not take account of deaths. In the hiatal hernia operation group up to 1 September, 2015, including among patients without one-year follow-up (*n* = 1.086) (Fig. 1), one death occurred in the axial (type I) hiatal hernia group (one out of 2792; 0.04%) and three deaths in the paraesophageal (types II–IV) group (three out of 1.333; 0.22%).

On one-year follow-up, no significant difference was identified in the recurrence rate or in the rates of pain at rest, on exertion or requiring treatment (Table 4). An additional analysis of patient outcome in relation to the individual hospital’s case load showed no significant differences for a case load of 1–49, 50–99, and ≥100 (Table 5).

**Table 5:** Outcome of patients depending on hospitals case load

	1–49 OPs		50–99 OPs		>100 OPs		
	*n*	%	*n*	%	*n*	%	*p*
Intraoperative complications							
Yes	51	3.46	10	2.56	40	3.39	0.701
No	1421	96.54	381	97.44	1140	96.61	
Postoperative complications (Clavien–Dindo classification grade I–III)							
Yes	24	1.63	8	2.05	12	1.02	0.199
No	1448	98.37	383	97.95	1168	98.98	
General complications							
Yes	63	4.28	10	2.56	48	4.07	0.299
No	1409	95.72	381	97.44	1132	95.93	
Recurrence on 1-year follow-up							
Yes	82	5.57	11	2.81	52	4.41	0.053
No	1390	94.43	380	97.19	1128	95.59	

### Multivariable analysis

#### Intraoperative complications

**Fig. 2:**
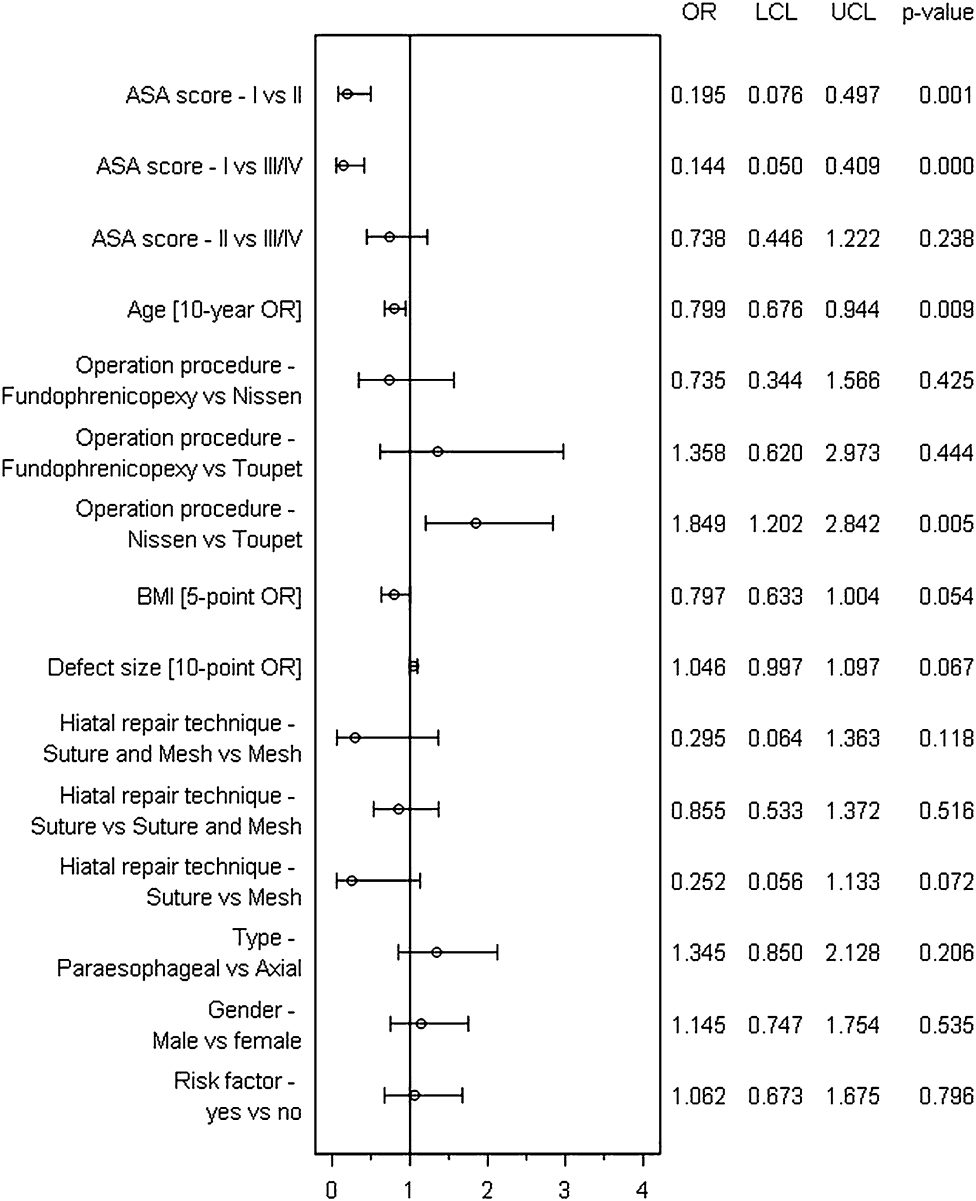
Forest plot: Multivariable analysis of influencing factors for intraoperative complications in hiatal hernia repair

The results of the model used for analysis of influencing factors for intraoperative complications are illustrated in Fig. 2 (model matching: *p* < 0.001). The risk of intraoperative complications was primarily influenced by the ASA score (*p* = 0.001). A lower ASA score (I vs II: 0.195 [0.076; 0.497]; I vs III/IV: 0.144 [0.050; 0.409] reduced the risk of intraoperative complications. Likewise, age and operative technique had a significant influence on the intraoperative complications. Accordingly, by comparison, a 10-year-older patient had a significantly lower intraoperative complication risk (10-year OR 0.799 [0.676; 0.944]). On the other hand, the complication risk was increased when the Nissen compared with the Toupet method was used (OR 1.849 [1.202; 2.842]; *p* = 0.005).

#### Surgical postoperative complications

Model matching for analysis of the postoperative complications, which reflects the suitability of the influence parameters to explain the outcome variable scores, was not significant (*p* = 0.335). As such, there was no evidence of the individual variables having significantly influenced the postoperative complication rate.

#### Complication-related reoperations

Model matching for complication-related reoperations, which reflects the suitability of the influence parameters to explain the outcome variable scores, was not significant (*p* = 0.249). As such, there was no evidence of the individual variables having significantly influenced the complication-related reoperation rate.

#### General postoperative complications

**Fig. 3:**
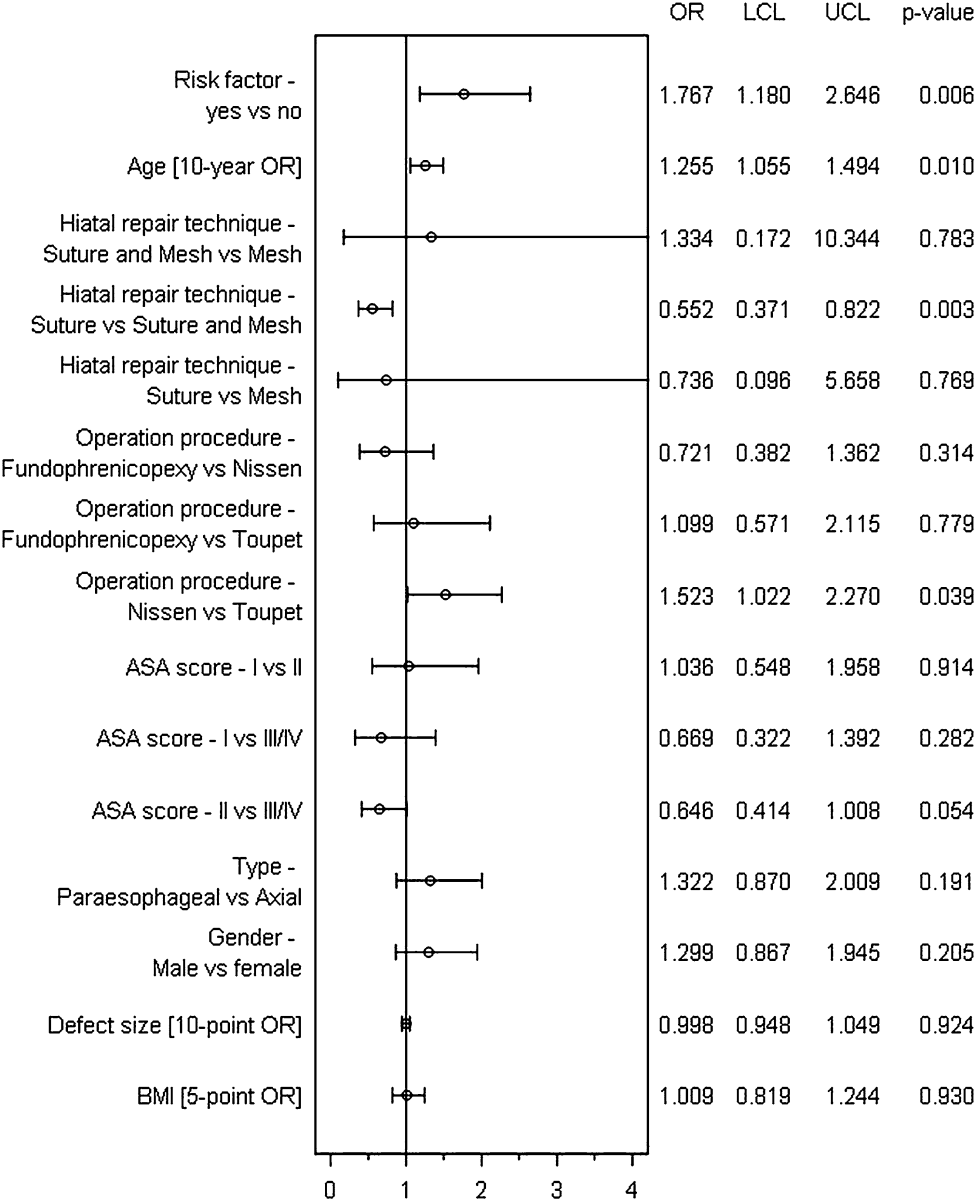
Forest plot: Multivariable analysis of influencing factors for general postoperative complications following hiatal hernia repair

The results of the model used for analysis of the general postoperative complication rate are shown in Fig. 3 (model matching: *p* < 0.001). Onset of general postoperative complications was primarily affected by the presence of risk factors (*p* = 0.006). The presence of at least one risk factor increased the general postoperative complication risk (OR 1.767 [1.180; 2.646]). Older patients, too, had an increased risk of general postoperative complications (10-year OR 1.255 [1.055; 1.494]). Conversely, the general postoperative complication risk was reduced in cases of hiatoplasty with suture alone compared with suture and mesh (OR 0.552 [0.371; 0.822]; *p* = 0.003).

### Recurrence on one-year follow-up

Model matching for recurrence on one-year follow-up, which reflects the suitability of the influence parameters to explain the outcome variable scores, was not significant (*p* = 0.180). As such, there was no evidence of the individual variables having significantly influenced the recurrence rate.

### Pain at rest on one-year follow-up

The results of the model used for analysis of pain at rest on one-year follow-up are summarized subsequently (model matching: *p* = 0.002). This was significantly impacted by risk factors, gender, and BMI. The rate was increased if there was at least one risk factor (OR 1.512 [1.135; 2.014]; *p* = 0.005). On the other hand, men (OR 0.664 [0.499; 0.864]; *p* = 0.005) and patients with higher BMI (5-point OR 0.821 [0.709; 0.951; *p* = 0.009) had a lower risk of pain at rest.

### Pain on exertion on follow-up

Model matching for pain on exertion on one-year follow-up, which reflects the suitability of the influence parameters to explain the outcome variable scores, was not significant (*p* = 0.154). As such, there was no evidence of the individual variables having significantly influenced the pain on exertion rate.

### Chronic pain requiring treatment on one-year follow-up

The results of the model used for analysis of chronic pain requiring treatment are summarized subsequently (model matching: *p* = 0.022). These, too, were significantly influenced by risk factors, gender, and BMI. The presence of at least one risk factor (OR 1.515 [1.119; 2.051]; *p* = 0.007) increased the risk of chronic pain requiring treatment. On the other hand, men (OR 0.712 [0.527; 0.961]; *p* = 0.026) and patients with higher BMI (5-point OR 0.839 [0.718; 0.981]; *p* = 0.028) had a lower risk of chronic pain requiring treatment.

## Discussion

This paper analyzes prospective data from the Herniamed Registry for 3043 patients with primary, elective, and laparoscopic repair of a hiatal hernia. Only patients with complete one-year follow-up results were included in the analysis. Since the outcome for patients with axial hiatal hernia and reflux disease differs greatly from that of patients with paraesophageal hiatal hernia, due to divergent patient characteristics and complexity of the repair technique, the two patient collectives were compared in the analysis presented here.

First of all, significant differences were noted in the patient characteristics. Patients with paraesophageal hernia were on average almost 10 years older, had a somewhat higher BMI, larger hernia defect, and tended more often to be female. The chief determinant for onset of significantly more perioperative complications among patients with paraesophageal hiatal hernia was a higher proportion of patients with ASA scores III/IV (34.8 vs 13.7%; *p* < 0.0001) and of patients with risk factors (30.8 vs 21.4%; *p* < 0.001).

Both these factors help to explain the significantly more frequent onset of general postoperative complications after repair of paraesophageal compared with axial hiatal hernias (6.0 vs 3.0%; *p* < 0.001). Multivariable analysis clearly demonstrates that the presence of at least one risk factor and higher age significantly increases the risk of general postoperative complications.

The greater complexity of the procedures used for paraesophageal hiatal hernia repair is reflected in a significantly higher intraoperative organ injury rate (3.7 vs 2.3%; *p* = 0.033) and significantly higher rate of complication-related reoperations (2.1 vs 1.1%; *p* = 0.033) compared with axial hiatal hernias.

The recurrence rate on one-year follow-up for patients after laparoscopic repair of axial hiatal hernias was 5.1% and for paraesophageal hiatal hernias it was 4.0% (*p* = 0.204), with the proportion of mesh-augmented hiatoplasties being significantly higher (35.2 vs 17.7%; *p* < 0.001) for paraesophageal hiatal hernias. The indication for mesh use was decided by the individual surgeon or hospital. The specific reasons for using a mesh were not documented.

Multivariable analysis did not find any evidence that the use of a mesh or other factors had a significant influence on the recurrence rate on one-year follow-up. That concords with the meta-analysis of four randomized controlled trials with 406 patients by Memom et al. [16]. It can only be speculated whether the significantly more frequent use of meshes for types II–IV hiatal hernias with highly significantly larger hiatal defects had led to a non-significant difference in the recurrence rate. In less than 1% of cases, only a mesh and no suture was used for hiatal closure, as reported in the literature [21]. That practice is not recommended in the guidelines [4].

There was no significant difference in the rates of pain at rest, pain on exertion, or pain requiring treatment on one-year follow-up between the patients after laparoscopic repair of axial (type I) vs paraesophageal (types II–IV) hiatal hernia.

Multivariable analysis demonstrates that the risk of pain at rest and pain requiring treatment was higher in the presence of risk factors, and was lower among men and in patients with higher BMI.

In summary, patients with elective laparoscopic repair of primary paraesophageal (types II–IV) vs axial (type I) hiatal hernia were found to have a significantly higher risk of general postoperative complications because of higher age and higher ASA score as well as the higher proportion of patients with at least one risk factor. Reflecting the greater complexity of laparoscopic paraesophageal (types II–IV) hiatal hernia repair procedures, there is greater likelihood of significantly more intraoperative organ injuries and postoperative complication-related reoperations. Accordingly, laparoscopic procedures for repair of paraesophageal (types II–IV) hiatal hernias should only be undertaken by experienced surgeons. Because of the higher risk of general postoperative complications, corresponding intensive medicine resources are needed.

## Herniamed Study Group

### Scientific Board

Köckerling, Ferdinand (Chairman) (Berlin); Bittner, Reinhard (Rottenburg); Fortelny, René (Wien); Jacob, Dietmar (Berlin); Koch, Andreas (Cottbus); Kraft, Barbara (Stuttgart); Kuthe, Andreas (Hannover); Lippert, Hans (Magdeburg): Lorenz, Ralph (Berlin); Mayer, Franz (Salzburg); Moesta, Kurt Thomas (Hannover); Niebuhr, Henning (Hamburg); Peiper, Christian (Hamm); Pross, Matthias (Berlin); Reinpold, Wolfgang (Hamburg); Simon, Thomas (Weinheim); Stechemesser, Bernd (Köln); Unger, Solveig (Chemnitz), Weyhe, Dirk (Oldenburg).

### Participants

Ahmetov, Azat (Saint-Petersburg); Alapatt, Terence Francis (Frankfurt/Main); Albayrak, Nurretin (Herne); Amann, Stefan (Neuendettelsau); Anders, Stefan (Berlin); Anderson, Jürina (Würzburg); Antoine, Dirk (Leverkusen); Apfelstedt, Heinrich (Solingen); Arndt, Anatoli (Elmshorn); Aschenbrenner, Michael (Spittal/Drau); Asperger, Walter (Halle); Avram, Iulian (Saarbrücken); Baikoglu-Endres, Corc (Weißenburg i. Bay.); Bandowsky, Boris (Damme); Barkus; Jörg (Velbert); Becker, Matthias (Freital); Behrend, Matthias (Deggendorf); Beuleke, Andrea (Burgwedel); Berger, Dieter (Baden-Baden); Birk, Dieter (Bietigheim-Bissingen); Bittner, Reinhard (Rottenburg); Blaha, Pavel (Zwiesel); Blumberg, Claus (Lübeck); Böckmann, Ulrich (Papenburg); Böhle, Arnd Steffen (Bremen); Bolle, Ludger (Berlin); Borchert, Erika (Grevenbroich); Born, Henry (Leipzig); Brabender, Jan (Köln); Breitenbuch von, Philipp (Radebeul); Brož, Miroslav (Ebersbach); Brückner, Torsten (Gießen); Brütting, Alfred (Erlangen); Buchert, Annette (Mallersdorf-Pfaffenberg; Buchholz, Torsten (Aurich; Budzier, Eckhard (Meldorf); Burchett, Bert (Teterow); Burghardt, Jens (Rüdersdorf); Cejnar, Stephan-Alexander (München); Chirikov, Ruslan (Dorsten); Claußnitzer, Christian (Ulm); Comman, Andreas (Bogen); Crescenti, Fabio (Verden/Aller); Daniels, Thies (Hamburg); Dapunt, Emanuela (Bruneck); Decker, Georg (Berlin); Demmel, Michael (Arnsberg); Descloux, Alexandre (Baden); Deusch, Klaus-Peter (Wiesbaden); Dick, Marcus (Neumünster); Dieterich, Klaus (Ditzingen); Dietz, Harald (Landshut); Dittmann, Michael (Northeim); Drummer, Bernhard (Forchheim); Eckermann, Oliver (Luckenwalde); Eckhoff, Jörn/Hamburg); Ehmann, Frank (Grünstadt); Eisenkrein, Alexander (Düren); Elger, Karlheinz (Germersheim); Engelhardt, Thomas (Erfurt); Erichsen, Axel (Friedrichshafen); Eucker, Dietmar (Bruderholz); Fackeldey, Volker (Kitzingen); Farke, Stefan (Delmenhorst); Faust, Hendrik (Emden); Federmann, Georg (Seehausen); Feichter, Albert (Wien); Fiedler, Michael (Eisenberg); Fikatas, Panagiotis (Berlin); Firl, Michaela (Perleberg); Fischer, Ines (Wiener Neustadt); Fleischer, Sabine (Dinslaken); Fortelny, René H. (Wien); Franczak, Andreas (Wien); Franke, Claus (Düsseldorf); Frankenberg von, Moritz (Salem); Frehner, Wolfgang (Ottobeuren); Friedhoff, Klaus (Andernach); Friedrich, Jürgen (Essen); Frings, Wolfram (Bonn); Fritsche, Ralf (Darmstadt); Frommhold, Klaus (Coesfeld); Frunder, Albrecht (Tübingen); Fuhrer, Günther (Reutlingen); Gassler, Harald (Villach); Gawad, Karim A. Frankfurt/Main); Gehrig, Tobias (Sinsheim); Gerdes, Martin (Ostercappeln); Germanov, German (Halberstadt; Gilg, Kai-Uwe (Hartmannsdorf); Glaubitz, Martin (Neumünster); Glauner-Goldschmidt, Kerstin (Werne); Glutig, Holger (Meissen); Gmeiner, Dietmar (Bad Dürrnberg); Göring, Herbert (München); Grebe, Werner (Rheda-Wiedenbrück); Grothe, Dirk (Melle); Günther, Thomas (Dresden); Gürtler, Thomas (Zürich); Hache, Helmer (Löbau); Hämmerle, Alexander (Bad Pyrmont); Haffner, Eugen (Hamm); Hain, Hans-Jürgen (Gross-Umstadt); Hammans, Sebastian (Lingen); Hampe, Carsten (Garbsen); Hanke, Stefan (Halle); Harrer, Petra (Starnberg); Hartung, Peter (Werne); Heinzmann, Bernd (Magdeburg); Heise, Joachim Wilfried (Stolberg); Heitland, Tim (München); Helbling, Christian (Uznach/Schweiz); Hellinger, Achim (Fulda); Hempen, Hans-Günther (Cloppenburg); Henneking, Klaus-Wilhelm (Bayreuth); Hennes, Norbert (Duisburg); Hermes, Wolfgang (Weyhe); Herrgesell, Holger (Berlin); Herzing, Holger Höchstadt); Hessler, Christian (Bingen); Heuer, Matthias (Herten); Hildebrand, Christiaan (Langenfeld); Höferlin, Andreas (Mainz); Hoffmann, Henry (Basel); Hoffmann, Michael (Kassel); Hofmann, Eva M. (Frankfurt/Main); Horbach, Thomas (Fürth); Hornung, Frederic (Wolfratshausen); Hudak, Attila (Suhl); Hübel-Abe, Jan (Ilmenau; Hügel, Omar (Hannover); Hüttemann, Martin (Oberhausen); Hüttenhain, Thomas (Mosbach); Hunkeler, Rolf (Zürich); Imdahl, Andreas (Heidenheim); Isemer, Friedrich-Eckart (Wiesbaden); Jablonski, Herbert Gustav (Sögel); Jacob, Dietmar (Berlin); Jansen-Winkeln, Boris (Leipig); Jantschulev, Methodi (Waren); Jenert, Burghard (Lichtenstein); Jugenheimer, Michael (Herrenberg); Junge, Karsten (Aachen); Junger, Marc (München); Kaaden, Stephan (Neustadt am Rübenberge); Käs, Stephan (Weiden); Kahraman, Orhan (Hamburg); Kaiser, Christian (Westerstede); Kaiser, Gernot Maximilian (Kamp-Lintfort); Kaiser, Stefan (Kleinmachnow); Kapischke, Matthias (Hamburg); Karch, Matthias (Eichstätt); Kasparek, Michael S. (München); Keck, Heinrich (Wolfenbüttel); Keller, Hans W. (Bonn); Kienzle, Ulrich (Karlsruhe); Kipfmüller, Brigitte (Köthen); Kirsch, Ulrike (Oranienburg); Klammer, Frank (Ahlen); Klatt, Richard (Hagen); Klein, Karl-Hermann (Burbach); Kleist, Sven (Berlin); Klobusicky, Pavol (Bad Kissingen); Kneifel, Thomas (Datteln); Knoop, Michael (Frankfurt/Oder); Knotter, Bianca (Mannheim); Koch, Andreas (Cottbus); Koch, Andreas (Münster); Köckerling, Ferdinand (Berlin); Köhler, Gernot (Linz); König, Oliver (Buchholz); Kornblum, Hans (Tübingen); Krämer, Dirk (Bad Zwischenahn); Kraft, Barbara (Stuttgart); Kratsch, Barthel (Dierdorf/Selters); Krausbeck, Matthias (Schwerin); Kreissl, Peter (Ebersberg); Krones, Carsten Johannes (Aachen); Kronhardt, Heinrich (Neustadt am Rübenberge); Kruse, Christinan (Aschaffenburg); Kube, Rainer (Cottbus); Kühlberg, Thomas (Berlin); Kühn, Gert (Freiberg); Kuhn, Roger (Gifhorn); Kusch, Eduard (Gütersloh); Kuthe, Andreas (Hannover); Ladberg, Ralf (Bremen); Ladra, Jürgen (Düren); Lahr-Eigen, Rolf (Potsdam); Lainka, Martin (Wattenscheid); Lammers, Bernhard J. (Neuss); Lancee, Steffen (Alsfeld); Lange, Claas (Berlin); Langer, Claus (Göttingen); Laps, Rainer (Ehringshausen); Larusson, Hannes Jon (Pinneberg); Lauschke, Holger (Duisburg); Lechner-Puschnig, Marina (Klagenfurt am Wörthersee/Österreich); Leher, Markus (Schärding); Leidl, Stefan (Waidhofen/Ybbs); Leisten, Edith (Köln); Lenz, Stefan (Berlin); Liedke, Marc Olaf (Heide); Lienert, Mark (Duisburg); Limberger, Andreas (Schrobenhausen); Limmer, Stefan (Würzburg); Locher, Martin (Kiel); Loghmanieh, Siawasch (Viersen); Lorenz, Ralph (Berlin); Luther, Stefan (Wipperfürth); Luyken, Walter (Sulzbach-Rosenberg); Mallmann, Bernhard (Krefeld); Manger, Regina (Schwabmünchen); Maurer, Stephan (Münster); May, Jens Peter (Schönebeck); Mayer, Franz (Salzburg); Mayer, Jens (Schwäbisch Gmünd); Mellert, Joachim (Höxter); Menzel, Ingo (Weimar); Meurer, Kirsten (Bochum); Meyer, Moritz (Ahaus); Mirow, Lutz (Kirchberg); Mittag-Bonsch, Martina (Crailsheim); Mittenzwey, Hans-Joachim (Berlin); Möbius, Ekkehard (Braunschweig), Mörder-Köttgen, Anja (Freiburg); Moesta, Kurt Thomas (Hannover); Moldenhauer, Ingolf (Braunschweig); Morkramer, Rolf (Radevormwald); Mosa, Tawfik (Merseburg); Müller, Hannes (Schlanders); Münzberg, Gregor (Berlin); Murr, Alfons (Vilshofen); Mussack, Thomas (St. Gallen); Nartschik, Peter (Quedlinburg); Nasifoglu, Bernd (Ehingen); Neumann, Jürgen (Haan); Neumeuer, Kai (Paderborn); Niebuhr, Henning (Hamburg); Nix, Carsten (Walsrode); Nölling, Anke (Burbach); Nostitz, Friedrich Zoltán (Mühlhausen); Obermaier, Straubing); Öz-Schmidt, Meryem (Hanau); Oldorf, Peter (Usingen); Olivieri, Manuel (Pforzheim); Passon, Marius (Freudenberg); Pawelzik, Marek (Hamburg); Pein, Tobias (Hameln); Peiper, Christian (Hamm); Peiper, Matthias (Essen); Pertl, Alexander (Spittal/Drau); Philipp, Mark (Rostock); Pickart, Lutz (Bad Langensalza); Pizzera, Christian (Graz); Pöllath, Martin (Sulzbach-Rosenberg); Pöschmann, Enrico (Thalwil); Possin, Ulrich (Laatzen); Prenzel, Klaus (Bad Neuenahr-Ahrweiler); Pröve, Florian (Goslar); Pronnet, Thomas (Fürstenfeldbruck); Pross, Matthias (Berlin); Puff, Johannes (Dinkelsbühl); Rabl, Anton (Passau); Raggi, Matthias Claudius (Stuttgart); Rapp, Martin (Neunkirchen); Reck, Thomas (Püttlingen); Reinpold, Wolfgang (Hamburg); Renter, Marc Alexander (Moers); Reuter, Christoph (Quakenbrück); Richter, Jörg (Winnenden); Riemann, Kerstin (Alzenau-Wasserlos); Riesener, Klaus-Peter (Marl); Rodehorst, Anette (Otterndorf); Roehr, Thomas (Rödental); Rössler, Michael (Rüdesheim am Rhein); Roncossek, Bremerhaven); Rosniatowski, Rolland (Marburg); Roth Hartmut (Nürnberg); Sardoschau, Nihad (Saarbrücken); Sauer, Gottfried (Rüsselsheim); Sauer, Jörg (Arnsberg); Seekamp, Axel (Freiburg); Seelig, Matthias (Bad Soden); Seidel, Hanka (Eschweiler); Seiler, Christoph Michael (Warendorf); Seltmann, Cornelia (Hachenburg); Senkal, Metin (Witten); Shamiyeh, Andreas (Linz); Shang, Edward (München); Siemssen, Björn (Berlin); Sievers, Dörte (Hamburg); Silbernik, Daniel (Bonn); Simon, Thomas (Weinheim); Sinn, Daniel (Olpe); Sinner, Guy (Merzig); Sinning, Frank (Nürnberg); Smaxwil, Constatin Aurel (Stuttgart); Sörensen, Björn (Lauf an der Pegnitz): Syga, Günter (Bayreuth); Schabel, Volker (Kirchheim/Teck); Schadd, Peter (Euskirchen); Schassen von, Christian (Hamburg); Schattenhofer, Thomas (Vilshofen); Scheidbach, Hubert (Neustadt/Saale); Schelp, Lothar (Wuppertal); Scherf, Alexander (Pforzheim); Scheuerlein, Hubert (Paderborn); Scheyer, Mathias (Bludenz); Schilling, André (Kamen); Schimmelpenning, Hendrik (Neustadt in Holstein); Schinkel, Svenja (Kempten); Schmid, Michael (Gera); Schmid, Thomas (Innsbruck); Schmidt, Ulf (Mechernich); Schmitz, Heiner (Jena); Schmitz, Ronald (Altenburg); Schöche, Jan (Borna); Schoenen, Detlef (Schwandorf); Schrittwieser, Rudolf (Bruck an der Mur); Schroll, Andreas (München); Schubert, Daniel (Saarbrücken); Schüder, Gerhard (Wertheim); Schürmann, Rainer (Steinfurt); Schultz, Christian (Bremen-Lesum); Schultz, Harald (Landstuhl); Schulze, Frank P. Mülheim an der Ruhr); Schulze, Thomas (Dessau-Roßlau); Schumacher, Franz-Josef (Oberhausen); Schwab, Robert (Koblenz); Schwandner, Thilo (Lich); Schwarz, Jochen Günter (Rottenburg); Schymatzek, Ulrich (Eitorf); Spangenberger, Wolfgang (Bergisch-Gladbach); Sperling, Peter (Montabaur); Staade, Katja (Düsseldorf); Staib, Ludger (Esslingen); Staikov, Plamen (Frankfurt am Main); Stamm, Ingrid (Heppenheim); Stark, Wolfgang (Roth); Stechemesser, Bernd (Köln); Steinhilper, Uz (München); Stengl, Wolfgang (Nürnberg); Stern, Oliver (Hamburg); Stöltzing, Oliver (Meißen); Stolte, Thomas (Mannheim); Stopinski, Jürgen (Schwalmstadt); Stratmann, Gerald (Goch); Straßburger, Harald (Alfeld); Stubbe, Hendrik (Güstrow/); Stülzebach, Carsten (Friedrichroda); Tepel, Jürgen (Osnabrück); Terzić, Alexander (Wildeshausen); Teske, Ulrich (Essen); Thasler, Wolfgang (München); Tichomirow, Alexej (Brühl); Tillenburg, Wolfgang (Marktheidenfeld); Timmermann, Wolfgang (Hagen); Tomov, Tsvetomir (Koblenz; Train, Stefan H. (Gronau); Trauzettel, Uwe (Plettenberg); Triechelt, Uwe (Langenhagen); Ulbricht, Wolfgang (Breitenbrunn); Ulcar, Heimo (Schwarzach im Pongau); Ungeheuer, Andreas (München); Unger, Solveig (Chemnitz); Verweel, Rainer (Hürth); Vogel, Ulrike (Berlin); Voigt, Rigo (Altenburg); Voit, Gerhard (Fürth); Volkers, Hans-Uwe (Norden); Volmer, Ulla (Berlin); Vossough, Alexander (Neuss); Wallasch, Andreas (Menden); Wallner, Axel (Lüdinghausen); Warscher, Manfred (Lienz); Warwas, Markus (Bonn); Weber, Jörg (Köln); Weber, Uwe (Eggenfelden); Weihrauch, Thomas (Ilmenau); Weiß, Johannes (Schwetzingen); Weißenbach, Peter (Neunkirchen); Werner, Uwe (Lübbecke-Rahden); Wessel, Ina (Duisburg); Weyhe, Dirk (Oldenburg); Wieber, Isabell (Köln); Wiens, Matthias (Affoltern); Wiesmann, Aloys (Rheine); Wiesner, Ingo (Halle); Withöft, Detlef (Neutraubling); Woehe, Fritz (Sanderhausen); Wolf, Claudio (Neuwied); Wolkersdörfer, Toralf (Pößneck); Yaksan, Arif (Wermeskirchen); Yildirim, Can (Lilienthal); Yildirim, Selcuk (Berlin); Zarras, Konstantinos (Düsseldorf); Zeller, Johannes (Waldshut-Tiengen); Zhorzel, Sven (Agatharied); Zuz, Gerhard (Leipzig).
